# Characterization of Nanoparticle Adsorption on Polydimethylsiloxane-Based Microchannels

**DOI:** 10.3390/s21061978

**Published:** 2021-03-11

**Authors:** Hirotada Hirama, Ryutaro Otahara, Shinya Kano, Masanori Hayase, Harutaka Mekaru

**Affiliations:** 1 Human Augmentation Research Center, National Institute of Advanced Industrial Science and Technology, Chiba 277-0882, Japan; shinya-kano@aist.go.jp (S.K.); h-mekaru@aist.go.jp (H.M.); 2 Faculty of Science and Technology, Tokyo University of Science, Chiba 278-8510, Japan; chikuzenni2826@gmail.com (R.O.); mhayase@rs.tus.ac.jp (M.H.)

**Keywords:** microfluidics, lab-on-a-chip, atomic force microscopy, exosomes, nanoparticles, adsorption, surface treatment

## Abstract

Nanoparticles (NPs) are used in various medicinal applications. Exosomes, bio-derived NPs, are promising biomarkers obtained through separation and concentration from body fluids. Polydimethylsiloxane (PDMS)-based microchannels are well-suited for precise handling of NPs, offering benefits such as high gas permeability and low cytotoxicity. However, the large specific surface area of NPs may result in nonspecific adsorption on the device substrate and thus cause sample loss. Therefore, an understanding of NP adsorption on microchannels is important for the operation of microfluidic devices used for NP handling. Herein, we characterized NP adsorption on PDMS-based substrates and microchannels by atomic force microscopy to correlate NP adsorptivity with the electrostatic interactions associated with NP and dispersion medium properties. When polystyrene NP dispersions were introduced into PDMS-based microchannels at a constant flow rate, the number of adsorbed NPs decreased with decreasing NP and microchannel zeta potentials (i.e., with increasing pH), which suggested that the electrostatic interaction between the microchannel and NPs enhanced their repulsion. When exosome dispersions were introduced into PDMS-based microchannels with different wettabilities at constant flow rates, exosome adsorption was dominated by electrostatic interactions. The findings obtained should facilitate the preconcentration, separation, and sensing of NPs by PDMS-based microfluidic devices.

## 1. Introduction

Given the growing popularity of nanoparticles (NPs) (e.g., poly(lactide-co-glycolide) NPs, solid lipid NPs, and exosomes) as diagnosis and analysis tools for pharmaceutical [1] and medical [2] applications, several methods for the detection and analysis of viruses [3] and nucleic acids [4] have been developed. Recently, a great deal of attention has been given to the study of exosomes, which contain proteins, lipids, and nucleic acids (including miRNAs) that are secreted by cells and are present in various body fluids such as urine [5], saliva [6], and blood [7]; exosomes also mediate communication between cells [8]. As exosome composition reflects the state of cells [9], it is expected to be a biomarker for diseases, such as cancer and dementia [8]. Thus, exosome-based diagnosis may be used to realize noninvasive or minimally invasive medicine [10]. In addition, exosomes, which can move between cells, are also promising as drug carriers in drug delivery systems (DDSs) [11].

Given that the physical properties of NPs depend on their size and surface charge [12,13], size- and charge-driven NP handling techniques are crucial for the detection and analysis of biomarkers such as exosomes, viral particles, and nucleic acids. As microchannel-based microfluidic devices allow for nano-, pico-, and femtoliter-scale manipulations [14] and are superior to rapid sample processing and analysis [15], microscale sample analysis [16], and high-throughput analysis [17], various NP separation and concentration techniques have been developed to increase NP detection sensitivity [13,18].

Polydimethylsiloxane (PDMS)-based microchannels are widely used for the handling and analysis of biological samples such as cells, exosomes, viral particles, and nucleic acids [5,13,19,20,21,22,23,24,25,26], offering advantages such as easy processability, high gas permeability, and low cytotoxicity [27]. PDMS-based microchannels for isolation and enrichment of exosomes [5,13,19,21] and for analysis of exosomes [20,22] have been widely developed. However, microchannels may nonspecifically adsorb nanoscale substances such as proteins and NPs [28,29] because of their large specific surface area [30], which may cause sample loss [23,30] and channel clogging [30,31,32,33]. In light of the above, NP adsorption behavior has been investigated for surfaces of solids such as mica and sapphire [34,35]. In a static environment with no flow, this behavior is affected by electrostatic properties, such as the Debye length [34] and zeta potential [35,36] of NPs, and the pH [37] and electrolyte concentration [33,35] of the dispersion medium. In contrast, adsorption behavior in a flow field is affected by the microchannel material [32], microchannel wettability [30], and hydrodynamic properties [30]. Therefore, the characterization of these effects in a microfluidic device with a flow field is vital for understanding NP adsorption.

Herein, we clarify the relationship between NP adsorptivity and the electrostatic interaction associated with NP and dispersion medium properties by characterizing NP adsorption on PDMS-based substrates and microchannels using atomic force microscopy (AFM) and negatively charged polystyrene NPs as models of exosomes, which are negatively charged in water [38]. NPs were dispersed in media with different pH and thus exhibited different zeta potentials. The main adsorption parameters were determined from the calculated NP–PDMS electrostatic interactions. Furthermore, the adsorption of exosomes on microchannels was probed by introducing exosome dispersions with different pH into microfluidic devices with different wettability (i.e., hydrophilicity/hydrophobicity).

## 2. Materials and Methods

### 2.1. Fabrication of PDMS-Based Substrate and Microfluidic Device

A PDMS-based substrate for polystyrene NP adsorption was fabricated as follows: the PDMS base was mixed for 1 min with the curing agent (Silpot 184, Dow Corning Toray Co., Ltd., Tokyo, Japan) in a 10:1 mass ratio, and the mixture (thickness: 2 mm) was poured into a polystyrene container, degassed, and cured at 80 °C for 2 h. After curing, the polymer was cut into a substrate with dimensions of 10 mm (length) × 5 mm (width) × 2 mm (thickness) and subjected to photo- and soft lithography [39] to fabricate a microfluidic device with a microchannel (30 mm long, 3 mm wide, and 100 µm deep) for polystyrene NP and exosome adsorption (as shown in Figure S1 in Supplementary Materials and as described below). A negative photoresist (SU-8 2100, KAYAKU Advanced Materials, Inc., Westborough, MA, USA) was spin-coated on a 4-inch silicon wafer and exposed to UV light through a photo mask, with subsequent development affording a 100-µm-thick convex mold. Finally, the pattern was transferred to PDMS to obtain a concave channel. Both hydrophobic (non-surface-treated) and hydrophilic (exposed to oxygen plasma for 30 s at a power of 100 W) microchannels were fabricated. A PDMS plate (with inlet and outlet holes fabricated by a biopsy punch) was placed on the fabricated microchannel for optimal adherence.

### 2.2. Preparation of NP Dispersions

Dispersions containing polystyrene NPs or exosomes were prepared. We used 100-nm and 250-nm polystyrene NPs (mean diameter = 124.3 ± 36.2 nm and 273.7 ± 32.2 nm, measured by dynamic light scattering, respectively; designed with carboxylic acid groups on the particle surface; Micromer^®^-redF, Micromod Partikeltechnologie GmbH, Rostock, Germany). To determine particle sizes, dynamic light scattering measurements were performed using an electrophoresis light-scattering spectrophotometer (ELS-Z2, Otsuka Electronics Co., Ltd., Osaka, Japan). The scattering angle of the measurement cell was 165°. Dispersions containing 100-nm and 250-nm polystyrene NPs at 7.6 × 10^10^ particles/mL and 7.5 × 10^10^ particles/mL, respectively, were prepared using 0.1 M phosphate buffered saline (PBS) solutions at pH 6, 7, or 8 (consisting of sodium dihydrogen phosphate and disodium hydrogen phosphate, as-purchased without the addition of acid or base to adjust pH; Fujifilm Wako Pure, Osaka, Japan). In this study, we used polystyrene NPs, which have a negative surface charge and size range similar to exosomes. A near-neutral pH was chosen to ensure compatibility with biomaterials. Dispersions containing exosomes (milk exosomes, mean diameter = 165.0 ± 2.0 nm, specification, Cosmo Bio Co., Ltd., Tokyo, Japan) at 7.6 × 10^10^ particles/mL were also prepared using 0.1 M PBS solutions at pH 6, 7, or 8. The pH of the body fluids that are the main source of exosomes (e.g., sweat, tears, blood, and urine) was considered when choosing the pH of the dispersion medium. Therefore, in this study, a slightly wider physiologically relevant pH range (i.e., pH 6–8) was investigated.

### 2.3. NP Adsorption Tests for PDMS Substrate and Microfluidic Device

**Figure 1:**
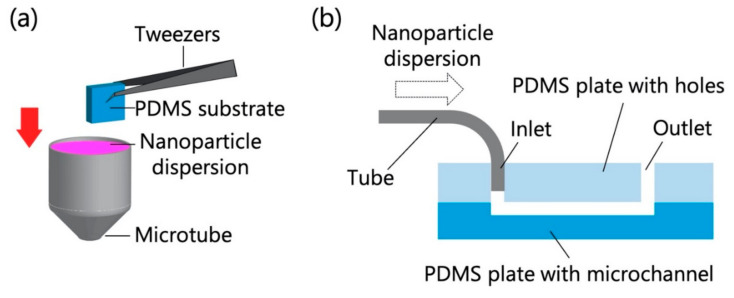
Schematics of particle adsorption tests for (**a**) the polydimethylsiloxane (PDMS)-based substrate and (**b**) the PDMS-based microchannel (30 mm long, 3 mm wide, and 100 µm deep).

The PDMS-based substrate was immersed in the chosen NP dispersion for 10 min (Figure 1a) and then rapidly dried by blowing off any remaining dispersion with compressed air. In another test, the microfluidic device and a syringe filled with the NP dispersion were connected with polytetrafluoroethylene tubes (inner diameter = 0.5 mm, outer diameter = 1.59 mm), and the NP dispersion was introduced into the device using a syringe pump (KDS100, KD Scientific, Holliston, MA, USA) at a constant flow rate (0.7 mL/h for polystyrene NPs; 0.7, 1.4, and 3.5 mL/h for exosomes) for 10 min (Figure 1b). In the experiments that used polystyrene NPs, the flow rate was fixed at 0.7 mL/h to compare the adsorptivity in static and dynamic environments, while in the experiments that used exosomes, the different flow rates were used to investigate the adsorptivity in dynamic environments with different flow rates. The flow rate range and temperature (20 °C) were determined on the basis of previous studies concerning the handling of exosomes using microchannels [5,21]. Because the ambient temperature in most laboratories is ~20 °C, this temperature was chosen for NP analysis. Subsequently, the PDMS-based microchannels were quickly peeled off, and their surfaces were promptly dried by blowing off any remaining dispersion with compressed air.

### 2.4. AFM Measurements

AFM (SFT-3500, Shimadzu Co. Ltd., Kyoto, Japan) was used in dynamic mode to observe the surfaces of PDMS-based substrates and PDMS-based microchannels with adsorbed NPs. For the observation of 250-nm polystyrene NPs, the scan area was set to 20 μm × 20 μm, while an area of 8 μm × 8 μm was used to observe 100-nm polystyrene NPs and exosomes. Five AFM images were randomly obtained per sample and analyzed using the ImageJ software (National Institutes of Health, Bethesda, MD, USA) to determine the area of the NPs in the scan area. The number of NPs adsorbed per unit area (*N*_ads_) was calculated as

(1) $$N_{\mathrm{ads}}=\frac{4}{\pi }\times \frac{\mathrm{Measured}\;\mathrm{particle}\;\mathrm{area}}{\mathrm{Scan}\;\mathrm{area}\;\times {\;\mathrm{Particle}\;\mathrm{diameter}}^{2}}$$

### 2.5. Zeta Potential Measurements

The zeta potentials of polystyrene NPs, exosomes, and PDMS-based substrates in 0.1 M PBS (pH 6, 7, or 8) were measured by laser Doppler electrophoresis using an electrophoresis light-scattering spectrophotometer (ELS-Z2, Otsuka Electronics Co., Ltd., Osaka, Japan). A measurement cell with a 1 mm deep gap was brought into close contact with the PDMS-based substrates to measure the zeta potentials. The apparent electrophoretic mobility of the monitored particles in the cell was then measured and the obtained values were analyzed to determine the zeta potential on the surface of the PDMS-based substrate at each pH. The measurement was performed once for each sample.

## 3. Results and Discussion

### 3.1. Polystyrene NP Adsorption on PDMS-Based Substrate

**Figure 2:**
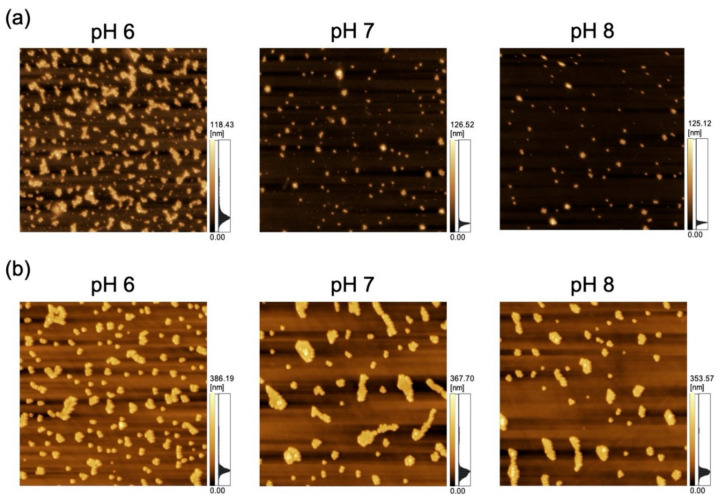
Atomic force microscopy (AFM) images of polydimethylsiloxane-based substrates immersed in variable-pH dispersions of (**a**) 100-nm nanoparticles (NPs; scan area = 8 μm × 8 μm) and (**b**) 250-nm NPs (scan area = 20 μm × 20 μm).

The adsorption of polystyrene NPs on the PDMS-based substrate was characterized by AFM imaging of the substrate that had been immersed in the corresponding NP dispersions (Figure 2).

**Figure 3:**
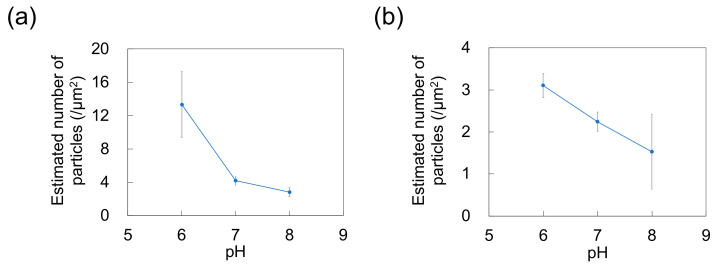
Effect of pH on the number of particles adsorbed on polydimethylsiloxane-based substrates immersed in dispersions of (**a**) 100-nm and (**b**) 250-nm nanoparticles (*n* = 5). Error bars indicate the standard deviation.

**Figure 4:**
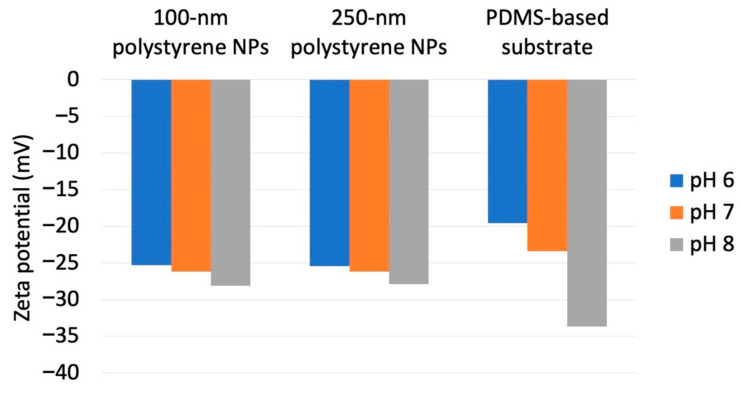
Zeta potentials obtained for different nanoparticle dimensions and dispersion medium pH.

As a previous study showed that *N*_ads_ is affected by zeta potential [35] and that NP adsorptivity is reduced by electrostatic repulsion due to substrate–particle and particle–particle interactions [40], we examined the effect of pH and zeta potential on *N*_ads_, revealing that this parameter decreased with increasing pH regardless of particle size (Figure 3; for comparison, adsorption on the PDMS-based substrate in the control medium without NPs is provided in Figure S1 in Supplementary Materials). Higher pH promoted deprotonation of carboxyl groups on the NP surface, thus resulting in greater negative charge and lower zeta potential (Figure 4). Lower zeta potentials at higher pH increase repulsions between the substrate and incoming particles (NPs to be adsorbed), and that between adsorbed particles (NPs previously adsorbed) and incoming particles, thus decreasing *N*_ads_.

**Figure 5:**
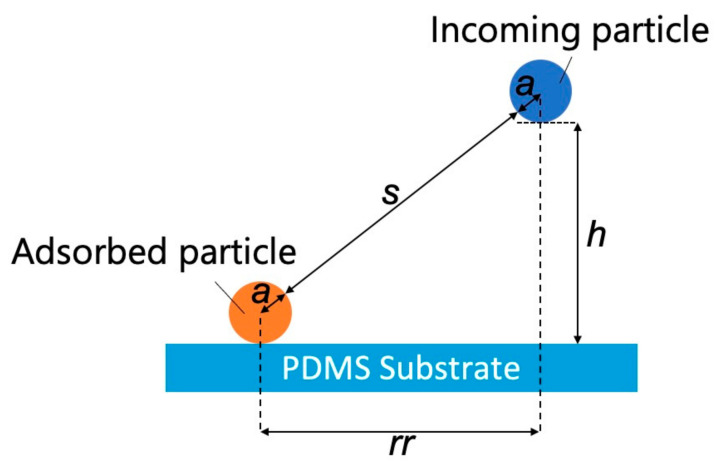
Model representing interactions between an adsorbed particle, an incoming particle, and a polydimethylsiloxane-based substrate (*s*: separation between the adsorbed and incoming particles, *rr*: projected center-to-center distance between the incoming particle and the adsorbed particle, *h*: separation between the incoming NP and the PDMS-based substrate, and *a*: particle radius).

We then examined the effects of the substrate–particle and particle–particle interaction energies using the model shown in Figure 5.

The separation (*s*) between the adsorbed and incoming particles can be expressed as

(2) $$s=\sqrt{rr^{2}+h^{2}}-2a$$

where *rr* is the projected center-to-center distance between the incoming particle and the adsorbed particle [36], *h* is the separation between the incoming NP and the PDMS-based substrate, and *a* is the particle radius. According to a previous study [36], the dimensionless electrostatic interaction energy (*ϕEL_pss_*) between the substrate surface and incoming particles can be expressed as

(3) $${\phi EL}_{pss}=\frac{16\varepsilon {\left(\frac{kT}{e}\right)}^{2}a\;\times \;\tanh\left(\frac{e\psi_{p}}{4kT}\right)\;\times \;\tanh\left(\frac{e\psi_{ss}}{4kT}\right)\;\times \;\exp\left(\frac{-h}{\kappa^{-1}}\right)}{kT}$$

where *ε* is the dielectric constant of the dispersion medium, *k* is the Boltzmann constant, *T* is the absolute temperature, *e* is the elementary charge, *ψ_p_* is the particle zeta potential, *ψ_ss_* is the zeta potential of the PDMS-based substrate surface, and *κ*^−1^ is the Debye length of the dispersion medium. The dimensionless electrostatic interaction energy between the adsorbed and incoming particles (*ϕEL_pp_*) can be expressed as follows [36,41]:

(4) $${\phi EL}_{pp}=\frac{{64\pi a\mathrm{kTn}}_{\infty }{\left(\kappa^{-1}\right)}^{2}\gamma^{2}\exp\left(\frac{-s}{\kappa^{-1}}\right)}{kT}$$

(5) $$\gamma =\tanh\left(\frac{ze\psi_{p}}{4kT}\right)=\frac{\exp\left(\frac{ze\psi_{p}}{2k\;T}\right)-1}{\exp\left(\frac{ze\psi_{p}}{2k\;T}\right)+1}$$

where *n_∞_* is the ionic concentration of the NP dispersion and *z* is the absolute value of the valence number. As these two interaction energies affect *N*_ads_ in the NP adsorption system employed in this study, the total interaction energy (*ϕEL_Total_*, which was made dimensionless) can be expressed as

(6) $${\phi EL}_{Total}={\phi EL}_{pss}+{\phi EL}_{pp}$$

**Figure 6:**
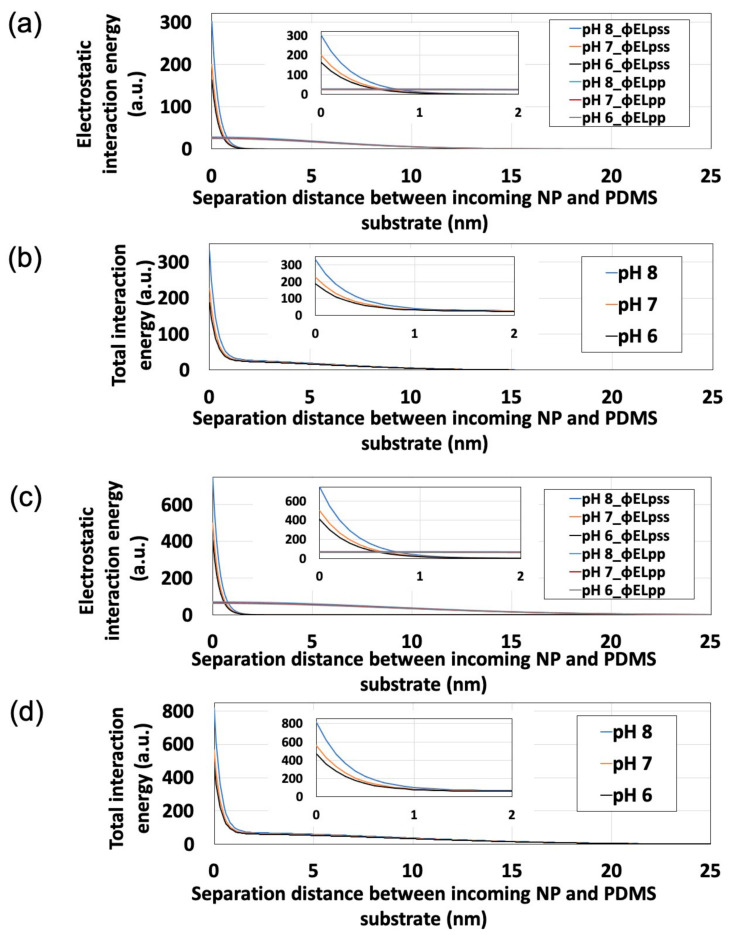
Effects of pH on interaction energy profiles obtained for polystyrene nanoparticles (NPs) approaching the polydimethylsiloxane-based substrate surface. Insets show the expanded origin regions. (**a**) *ϕEL_pss_* and *ϕEL_pp_* for 100-nm polystyrene NPs. (**b**) *ϕEL_Total_* for 100-nm polystyrene NPs. (**c**) *ϕEL_pss_* and *ϕEL_pp_* for 250-nm polystyrene NPs. (**d**) *ϕEL_Total_* for 250-nm polystyrene NPs.

Substitution of polystyrene NP zeta potentials (Figure 4) into Equations (3)–(6) allowed us to calculate *ϕEL_pss_*, *ϕEL_pp_*, and *ϕEL_Total_* (Figure 6). Here, *ε*, *T*, and *n_∞_* were estimated as 78.3, 293 K, and 1000 *N_a_ × C* (*N_a_* = Avogadro’s constant; *C* = electrolyte concentration of dispersion medium, mol/L), respectively. Additionally, to simplify the calculation, we estimated *κ*^−1^ and *z* as 0.64 nm and 3, respectively, assuming the PBS dispersion medium to be an aqueous solution of a trivalent symmetric electrolyte. For this estimation, assuming that 50% of each monovalent dihydrogen phosphate ion and divalent hydrogen phosphate ion are present in the dispersion medium near neutrality, *z* was set to 1.5 (=1 × 0.5 + 2 × 0.5) and *κ*^−1^ was determined using the Debye-Hückel parameter [42]:

(7) $$\kappa ={\left(\frac{2n\infty z^{2}e^{2}}{\varepsilon_{r}\varepsilon_{0}k\;T}\right)}^{\frac{1}{2}}$$

where *ε_r_* and *ε_0_* are the relative permittivity of the liquid (78.3) and the permittivity of a vacuum (8.85 × 10^−12^ F·m^−1^). Here, the maximum value of *ϕEL_pp_* was determined as the point at which the particles come into contact with each other (i.e., at *rr*/*a* = 2). With decreasing separation between an incoming NP and the PDMS-based substrate (i.e., as the particle approaches the substrate), *ϕEL_Total_* increases regardless of particle size. At a certain point away from the substrate, *ϕEL_pp_* exceeds *ϕEL_pss_*, therefore being the dominant influence, whereas *ϕEL_pss_* exceeded *ϕEL_pp_* in the vicinity of the substrate, thus dominating under these conditions. Furthermore, in the vicinity of the substrate, *ϕEL_pss_* remarkably increases with increasing pH, while *ϕEL_pp_* remains unaffected by changes in pH. These results suggest that the behavior of this NP adsorption system is largely determined by *ϕEL_pss_*, and that pH can control electrostatics to influence *ϕEL_pss_*, *ϕEL_Total_*, and NP adsorptivity.

The zeta potential of PDMS can change depending on the mixing time of the PDMS prepolymer (Table S1 in Supplementary Materials). Therefore, it may be possible to control adsorptivity by changing other PDMS synthetic parameters in addition to the mixing time. The PDMS base was mixed with the curing agent in a 10:1 mass ratio, which was previously reported as an appropriate ratio for fabricating microfluidic structures [43]. Qiang et al. reported that PDMS tends to swell more as the ratio of the base to the curing agent increases [44]. Furthermore, Oliveira et al. reported that more the swelling of the polymer occurs (i.e., chitosan in their study), the greater the zeta potential [45]. Therefore, the zeta potential of PDMS may also increase as the ratio of the base to the curing agent increases. Moreover, because zeta potential is affected by temperature [46,47], a large temperature change can affect adsorption because zeta potential changes.

The effect of particle size on *N*_ads_ was also investigated. In the pH range studied (pH 6–8), the *N*_ads_ of the 250-nm NPs remained lower than that of the 100-nm NPs (Figure S2 in Supplementary Materials). This adsorptivity trend is supported by the *ϕEL_Total_* generated in the vicinity of the PDMS-based substrate, which is smaller for 100-nm NPs than for 250-nm NPs (as shown in Figure 6b,d).

### 3.2. Polystyrene NP Adsorption on PDMS-Based Microchannels

**Figure 7:**
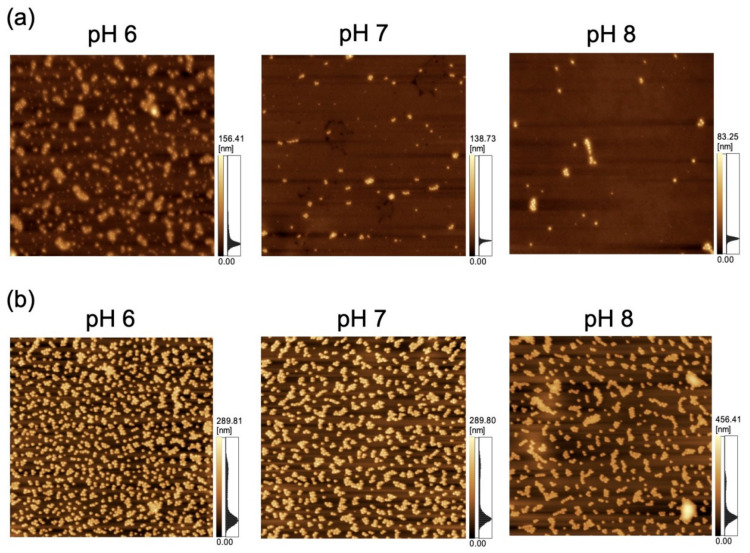
Atomic force microscopy images of a polydimethylsiloxane-based microchannel exposed to variable-pH dispersions of (**a**) 100-nm (scan area = 8 μm × 8 μm) and (**b**) 250-nm nanoparticles (scan area = 20 μm × 20 μm) at a flow rate of 0.7 mL/h.

**Figure 8:**
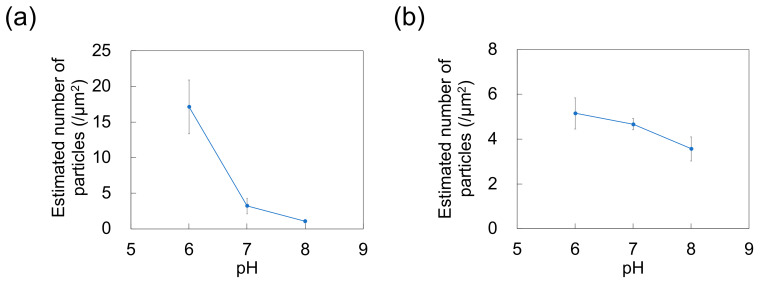
Effect of pH on the number of particles adsorbed on polydimethylsiloxane-based microchannels exposed to dispersions of (**a**) 100-nm and (**b**) 250-nm nanoparticles (*n* = 5) at a flow rate of 0.7 mL/h. Error bars represent the standard deviation.

The adsorption of NPs onto PDMS-based microchannels was probed by AFM imaging of the surface of the microchannels after exposure to polystyrene NP dispersions supplied at a constant flow rate of 0.7 mL/h (Figure 7). At each pH, the polystyrene NPs were adsorbed on the PDMS-based microchannels. *N*_ads_ was calculated by substituting the surface coverage ratio (determined by analysis of AFM images) into Equation (1) (Figure 8). As in the case of adsorption on the PDMS-based substrate, *N*_ads_ decreases with increasing pH (i.e., with decreasing zeta potential of the NPs and the PDMS-based microchannel wall) regardless of particle size. This suggests that repulsions between the substrate and NPs increase (and thus, the number of adsorbed NPs decreases) even in the flow field.

### 3.3. Exosome Adsorption on PDMS-Based Microchannels

**Figure 9:**
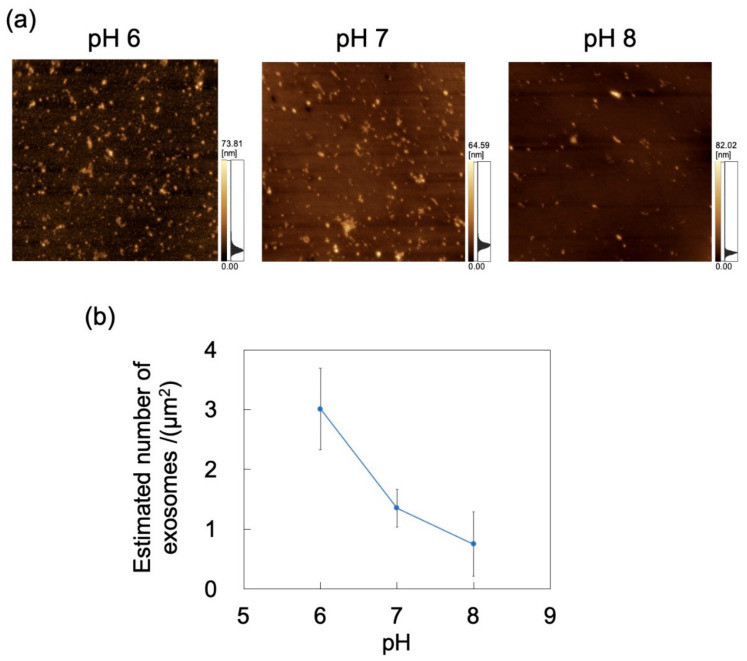
Adsorption of exosomes on a PDMS microchannel exposed to exosome dispersions with different pH at a constant flow rate of 0.7 mL/h. (**a**) Atomic force microscopy images of exposed polydimethylsiloxane (PDMS)-based microchannels. Scan area = 8 μm × 8 μm. (**b**) Number of exosomes adsorbed on PDMS-based microchannel (*n* = 5) as a function of pH. Error bars represent the standard deviation.

The adsorption of exosomes on PDMS-based microchannels was probed by AFM imaging of the surface of these channels exposed to exosome dispersions that were introduced at a constant flow rate of 0.7 mL/h (Figure 9a). *N*_ads_ for exosomes was calculated by substituting the surface coverage ratio (determined by analysis of AFM images) into Equation (1) (Figure 9b). As for polystyrene NPs, *N*_ads_ decreases with increasing pH (and hence, with decreasing zeta potential) of the dispersion medium. With increasing pH, the extent of deprotonation of amino and carboxyl groups of the membrane proteins on the exosome surface increases, thereby increasing surface charge (represented by zeta potential; Table 1), consistent with a previous report [23]. In turn, lower zeta potentials enhance repulsions between the microchannel and the incoming particles, and between the particles adsorbed on the microchannel wall and the incoming particles, thus further decreasing *N*_ads_. This suggests that the zeta potential decrease associated with both the microchannel and the exosomes hinders the adsorption of NPs on PDMS. In contrast to the PDMS-based substrate, no clear effect of particle size on *N*_ads_ was observed in the PDMS-based microchannels (Figure S3 in Supplementary Materials).

**Figure 10:**
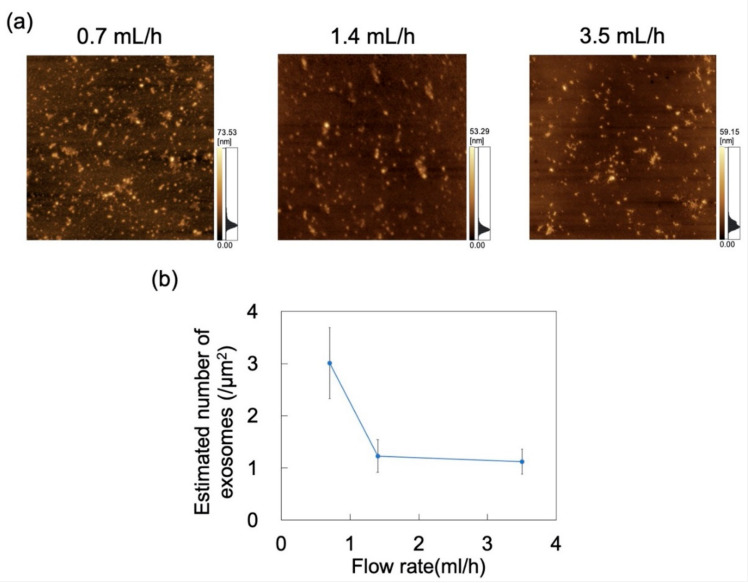
Adsorption of exosomes on polydimethylsiloxane (PDMS)-based microchannels at pH 6 and flow rates of 0.7, 1.4, and 3.5 mL/h. (**a**) AFM images of PDMS-based microchannels. Scan area = 8 μm × 8 μm. (**b**) Number of exosomes adsorbed on PDMS-based microchannels (*n* = 5) as a function of flow rate. Error bars represent the standard deviation.

The effect of flow rate (0.7, 1.4, and 3.5 mL/h) on the adsorption of exosomes at constant pH was probed by AFM imaging of the surface of the PDMS microchannels exposed to exosome dispersions (Figure 10a). *N*_ads_ was determined from AFM images as described in Section 2.4, decreasing with increasing flow rate (Figure 10b). The effect of shear stress in the flow field was then examined. For a Newtonian fluid, shear stress (*τ*) in a laminar flow field is given by

(8) $$\tau =\eta \gamma_{shear}$$

where *η* is the viscosity and *γ_shear_* is the shear rate that (in the microchannel) is proportional to flow rate [48]. Maintaining constant microchannel dimensions and dispersion medium gives rise to shear stress in the flow field that is proportional to the flow rate. These results suggest that exosome adsorption on the microchannels can be suppressed by increasing the flow rate to in turn increase the shear stress acting on the exosomes. Conversely, *N*_ads_ can increase at lower flow rates.

**Figure 11:**
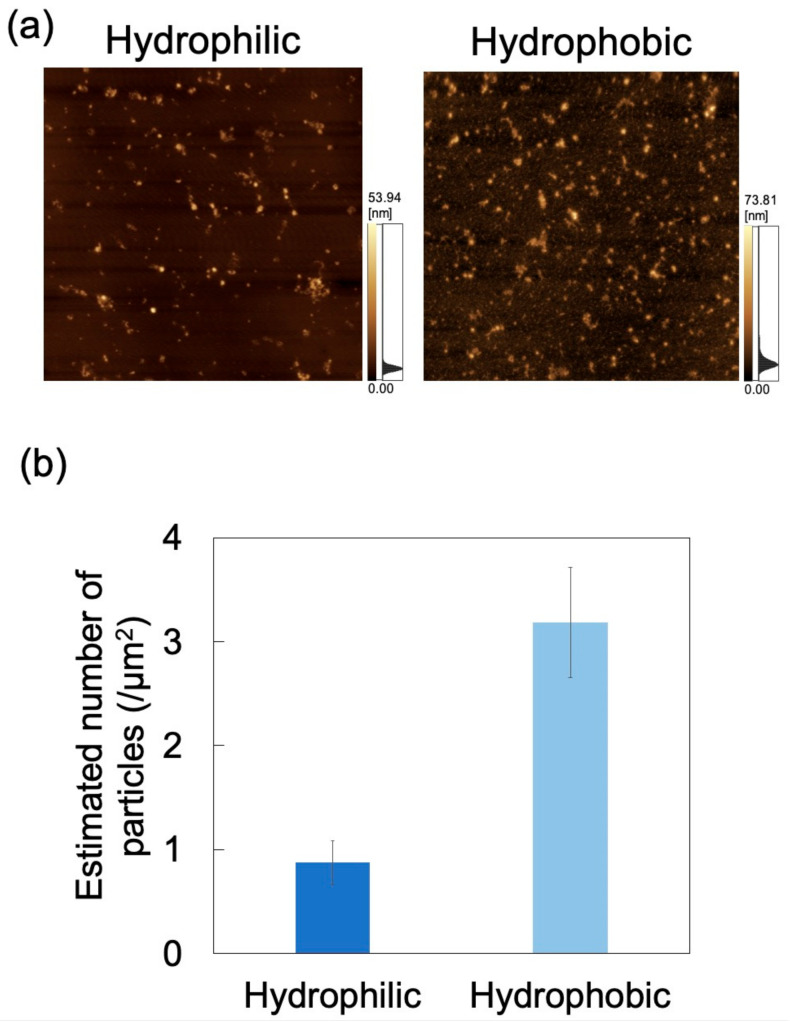
Adsorption of exosomes on hydrophilic and hydrophobic polydimethylsiloxane (PDMS)-based microchannels at pH 6 and 0.7 mL/h. (**a**) Atomic force microscopy images of PDMS-based microchannels with different wettability. Scan area = 8 μm × 8 μm. (**b**) Number of exosomes adsorbed on PDMS-based microchannels (*n* = 5). Error bars represent the standard deviation.

For applications involving biological samples, the inner wall of the microchannel is subjected to surface treatment to suppress adsorption of the target substance [49]. Herein, microchannel wettability (i.e., hydrophilicity/hydrophobicity) was modified via a physical treatment method (i.e., oxygen plasma treatment) rather than a chemical surface treatment method. In order to eliminate the temporal change of hydrophilicity obtained by plasma treatment as much as possible, the dispersion was brought into contact with the microchannel immediately (within 5 min) after plasma treatment. The zeta potential of PDMS treated with oxygen plasma is −59.97 mV (in deionized water, 0.5–1 h after treatment) [50]. The effect of wettability on exosome adsorption was investigated using AFM imaging of PDMS-based microchannels (either hydrophilic or hydrophobic), exposed to an exosome dispersion at pH 6 and a constant flow rate of 0.7 mL/h (Figure 11a). *N*_ads_ was determined from AFM images as described above (Figure 11b) and was smaller for hydrophilic microchannels than for hydrophobic ones. Oxygen plasma treatment of PDMS-based microchannels is known to produce silanol groups on the channel surface, resulting in its hydrophilization [51,52]. As hydroxyl groups are negatively charged in water, the PDMS surface in contact with the NP dispersion medium can attain a larger negative charge to increase repulsions between exosomes and PDMS, and hence reduce *N*_ads_. This suggests that exosome adsorptivity can be controlled by the zeta potential and wettability of the microchannel surface. Additionally, the surface of PDMS hydrophilized by plasma treatment remains hydrophilic upon immersion in water [53]. Therefore, hydrophilicity can be maintained while reducing NP adsorption by filling the microchannels with pure water until the dispersion is introduced into the microchannels after plasma treatment. Future investigations will involve measuring the zeta potential of the PDMS substrate after plasma treatment and comparing it with the zeta potential of the PDMS substrate before plasma treatment in order to provide a more quantitative assessment to clarify the relationship between wettability and adsorptivity.

## 4. Conclusions

Herein, we investigated the relationship between NP adsorptivity and electrostatic interactions associated with the NPs and dispersion medium properties by probing NP adsorption on PDMS-based microchannels using AFM. The adsorptivity of negatively charged polystyrene NPs (used as exosome models) on PDMS-based microchannels increased with increasing pH (i.e., with decreasing zeta potential of NPs and PDMS-based microchannels) of the NP dispersion. This indicates that the adsorption of polystyrene NPs on PDMS-based microchannels can be controlled by the electrostatic interactions between NPs and the microchannel. Furthermore, we investigated the adsorption of exosomes on PDMS-based microchannels in a flow field, thus demonstrating that these biological NPs behave similarly to polystyrene NPs. Moreover, hydrophilic microchannels can be prepared in order to reduce sample loss of NPs similar to exosomes by employing a relatively high flow rate and a slightly alkaline pH environment. As surface roughness of the substrate can contribute to particle adsorption, future studies will address how this affects adsorptivity. From the viewpoint of particle surface science, the behavior of simulated body fluids other than PBS must be investigated over a wider range of pH values, which is the subject of future research. Positively charged NPs can also be investigated as model particles for exosomes to further delineate our understanding of exosomes, which are the complex vesicles comprised of proteins, nucleic acids, and lipids, and NP adsorption phenomena. The findings obtained in the current study are expected to facilitate the pre-concentration, separation, and sensing of NPs using PDMS-based microfluidic devices.

## Figures and Tables

**Table 1:** Effect of pH on the zeta potentials of exosomes.

pH	Zeta Potential (mV)
6	−14.8
7	−16.8
8	−17.3

## Supplementary Materials

The following are available online at https://www.mdpi.com/1424-8220/21/6/1978/s1, Figure S1: Control tests for adsorption of nanoparticles on PDMS-based substrates, Figure S2: Effect of particle size on the number of particles adsorbed on PDMS-based substrates, Figure S3: Effect of particle size on the number of particles adsorbed on PDMS-based microchannels, Table S1: Effect of mixing time on the zeta potentials of PDMS-based substrates.
