# Supplementary material for: Characterization of Nanoparticle Adsorption on Polydimethylsiloxane-Based Microchannels
Source: Sensors (Basel). 2021 Mar 11;21(6):1978. doi: 10.3390/s21061978 (PMC7998103; doi:10.3390/s21061978)
Supplement: Supplementary file 1 [file sensors-21-01978-s001.pdf]

sensors

Supplementary Materials

Characterization of Nanoparticle Adsorption on Polydimethylsiloxane-Based Microchannels

Hirotada Hirama [1,*,†], Ryutaro Otahara [2,†], Shinya Kano [1], Masanori Hayase [2], Harutaka Mekaru [1]

[1] Human Augmentation Research Center, National Institute of Advanced Industrial Science and Technology, Chiba 277-0882, Japan; shinya-kano@aist.go.jp (S.K.); h-mekaru@aist.go.jp (H.M.)

[2] Faculty of Science and Technology, Tokyo University of Science, Chiba 278-8510, Japan; chikuzenni2826@gmail.com (R.O.); mhayase@rs.tus.ac.jp (M.H.)

* Correspondence: h.hirama@aist.go.jp; Tel.: +81-29-861-3065

† These authors contributed equally to this work.

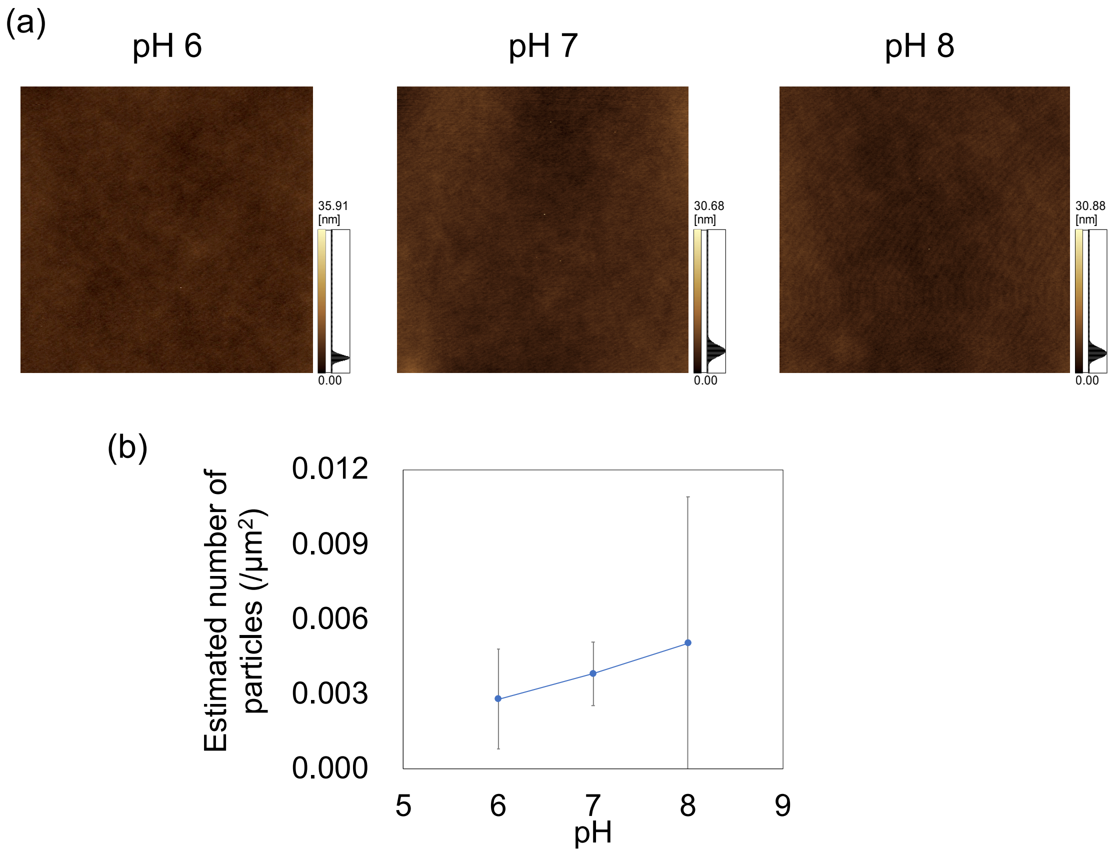

Figure S1. Control tests for adsorption of nanoparticles on polydimethylsiloxane (PDMS)-based substrates. (a) AFM images of PDMS-based substrates immersed in medium without nanoparticles. Scan area = 20 μm × 20 μm. (b) Number of nanoparticles adsorbed on the PDMS-based substrates (n = 5) as a function of pH.

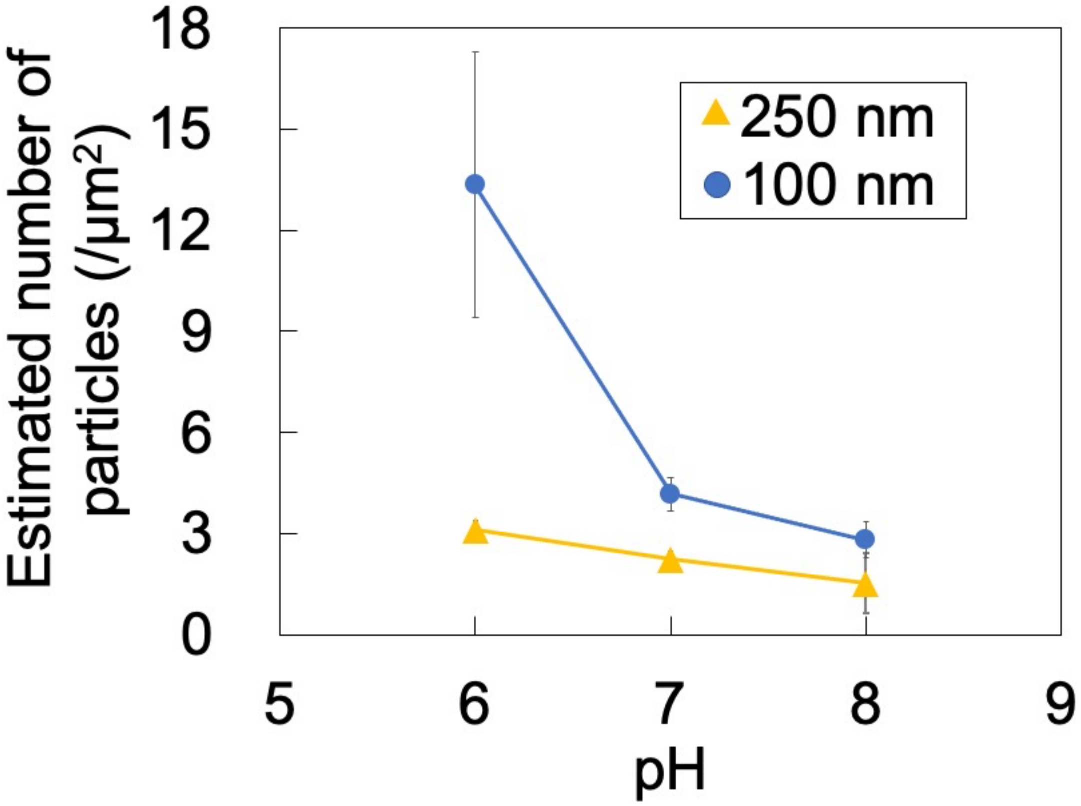

Figure S2. Effect of particle size on the number of particles adsorbed on polydimethylsiloxane-based substrates immersed in dispersions of nanoparticles (*n* = 5). Error bars represent the standard deviation.

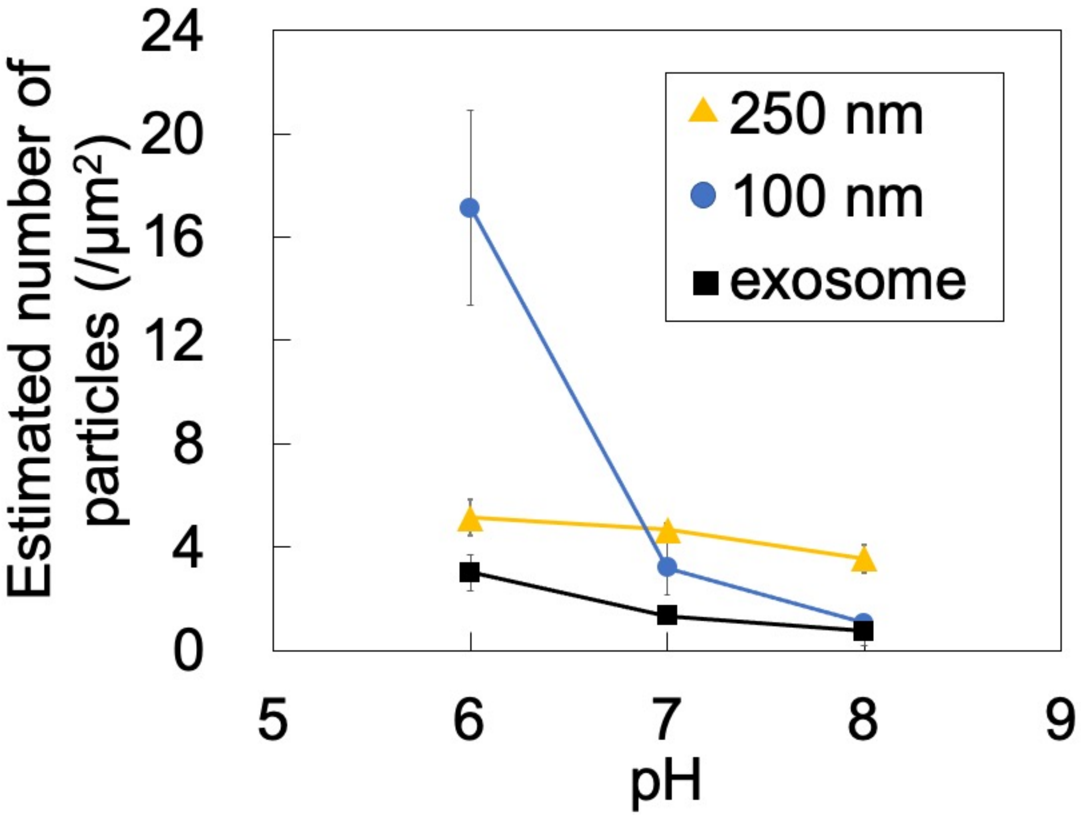

Figure S3. Effect of particle size on the number of particles adsorbed on polydimethylsiloxane-based microchannels exposed to dispersions of nanoparticles (n = 5) at a flow rate of 0.7 mL/h. Error bars represent the standard deviation.

Table S1. Effect of PDMS mixing time on the zeta potentials of PDMS-based substrates immersed in PBS medium at pH 6.

Mixing time (min)	Zeta potential (mV)
5	−20.0
10	−16.0
20	−16.0
